# The *N-*Glycosylation of Mouse Immunoglobulin G (IgG)-Fragment Crystallizable Differs Between IgG Subclasses and Strains

**DOI:** 10.3389/fimmu.2017.00608

**Published:** 2017-05-31

**Authors:** Noortje de Haan, Karli R. Reiding, Jasminka Krištić, Agnes L. Hipgrave Ederveen, Gordan Lauc, Manfred Wuhrer

**Affiliations:** ^1^Center for Proteomics and Metabolomics, Leiden University Medical Center, Leiden, Netherlands; ^2^Glycoscience Research Laboratory, Genos, Zagreb, Croatia

**Keywords:** mouse, immunoglobulin G, Fc-glycosylation, *N*-glycans, glycopeptides, LC-MS, MALDI-TOF-MS, HILIC-UPLC-fluorescence

## Abstract

*N*-linked glycosylation of the fragment crystallizable (Fc)-region of immunoglobulin G (IgG) is known to have a large influence on the activity of the antibody, an effect reported to be IgG subclass specific. This situation applies both to humans and mice. The mouse is often used as experimental animal model to study the effects of Fc-glycosylation on IgG effector functions, and results are not uncommonly translated back to the human situation. However, while human IgG Fc-glycosylation has been extensively characterized in both health and disease, this is not the case for mice. To characterize the glycosylation profile of murine IgG-Fc and in addition evaluate the systematic glycosylation differences between mouse strains, sexes, and IgG subclasses, we used nanoliquid chromatography mass spectrometry (nanoLC-MS(/MS)) to look at the subclass-specific IgG Fc-glycopeptides of male and female mice from the strains BALB/c, C57BL/6, CD-1, and Swiss Webster. The structural analysis revealed the presence of predominantly fucosylated, diantennary glycans, with varying amounts of galactosylation and α2,6-sialylation. In addition, we report glycosylation features not previously reported in an Fc-specific way on murine IgG, including monoantennary, hybrid, and high mannose structures, as well as diantennary structures without a core fucose, with a bisecting *N-*acetylglucosamine, or with α1,3-galactosylation. Pronounced differences were detected between strains and the IgG subclasses within each strain. Especially the large spread in galactosylation and sialylation levels found between both strains and subclasses may vastly influence IgG effector functions. Mouse strain-based and subclass-specific glycosylation differences should be taken into account when designing and interpreting immunological and glycobiological mouse studies involving IgG effector functions.

## Introduction

Immunoglobulin G (IgG) is the most abundant antibody in human plasma and plays a crucial role in the humoral immune response (1). Various effector functions of IgG are affected by its fragment crystallizable (Fc)-glycosylation, which has shown to influence the binding of IgG-Fc to, e.g., Fcγ-receptors (FcγRs) and C-type lectins (2–4). Changes in IgG Fc-glycosylation are associated with various diseases and physiological processes. For example, galactosylation on total IgG is decreased in rheumatoid arthritis and active tuberculosis infections, and increases with pregnancy (5–7). Fucosylation on the other hand, is decreased on alloantibodies against red blood cells and platelets as well as on gp120-specific antibodies in HIV-infected patients (8–10). Modification of IgG Fc-glycosylation has also shown to be a suitable measure to improve the efficacy of therapeutic monoclonal antibodies (11).

Mice are often used as experimental animal models to study the effects of Fc-glycosylation on IgG effector functions (2, 12). Various mouse strains are established for different research areas, for example outbred strains like CD-1 or Swiss Webster are used frequently for toxicological and pharmaceutical studies (13). In addition, because CD-1 mice are efficient breeders, they are regularly used in genetic experiments (14). On the other hand, inbred strains like BALB/c and C57BL/6 are often used to study infectious diseases and cancer (15, 16).

Contrary to the human IgG Fc-glycosylation, mice predominantly express the sialic acid *N*-glycolylneuraminic acid (Neu5Gc), while humans exclusively express *N*-acetylneuraminic acid (Neu5Ac) (17). Furthermore, as IgG Fc-glycosylation is partly genetically determined (18) and partly influenced by environmental factors like exposure to immunological challenges (19), baseline Fc-glycosylation may play a role in the outcome of an immunological study, and may confound the potential translation to the human situation. Differences in total plasma *N*-glycosylation between mouse strains were demonstrated before (20). Interestingly, glycans that are for humans known to be predominantly derived from IgG (21) showed both sex and strain specific differences in the murine total plasma *N*-glycome study. For example, galactosylation of diantennary fucosylated species, which is a known immune modulator on IgGs (4), was reported to be higher for BALB/c and C57BL/6 mice, when compared with CD-1 and Swiss Webster (20).

In contrast to human IgG, which can be divided into four subclasses (IgG1–4), murine IgG only knows three subclasses (IgG1–3) (22). In addition, murine IgG2 can be split in the isotypes IgG2a, 2b and 2c, of which IgG2a and 2c are allelic variants and further sequence variants are known for IgG1 and IgG2b (23, 24). Like for human IgG, the affinity of murine IgG for the various FcγRs differs per subclass (22, 25–27). For example, defucosylation of IgG Fc-glycans has been found to improve the FcγR-mediated pro-inflammatory activity of murine IgG2b, while this is not the case for murine IgG1 (28). Despite its importance and some previous descriptions of single case murine IgG glycosylation (23), no comprehensive strain- and subclass-specific characterization was hitherto performed.

We used nanoliquid chromatography (nanoLC) coupled to electrospray ionization (ESI)-mass spectrometry (MS) to analyze glycopeptides derived from murine IgG. In this way, we were able to separate the *N*-glycosylation present on IgG1 and its sequence variant (IgG1i), as well as on IgG2b, IgG2a/c, and IgG3, and to perform a subclass-specific relative quantification thereof. The glycosylation profiling of mice from each of the commonly used strains BALB/c, C57BL/6, CD-1, and Swiss Webster revealed significant differences between both the mouse strains and the subclasses within the strains. In addition, nanoLC-MS/MS allowed the characterization of glycan structures which, to our knowledge, were not previously reported on polyclonal murine IgG-Fc. These included high-mannose structures, hybrid structures, and structures with a bisecting *N-*acetylglucosamine (GlcNAc), without a core fucose, or with α1,3-linked galactose attached to the β-linked-galactose. Next to Neu5Gc, we detected Neu5Ac on several of the subclasses. Finally, previously reported matrix-assisted laser desorption/ionization (MALDI)-time-of-flight (TOF)-MS(/MS) (20, 29) and ultraperformance liquid chromatography (UPLC)-fluorescence (30) (Krištić et al., manuscript submitted) methods allowed to differentiate between, respectively, sialic acid linkages and antenna galactosylation in monogalactosylated species.

## Materials and Methods

### Chemicals

Ultrapure deionized water was generated by the Purelab Ultra, maintained at ≥18.2 MΩ (Veolia Water Technologies Netherlands B.V., Ede, the Netherlands) and used throughout. Disodium hydrogen phosphate dihydrate (Na_2_HPO_4_⋅2H_2_O), potassium dihydrogen phosphate (KH_2_PO_4_), NaCl, sodium dodecyl sulfate (SDS), ethanol, glacial acetic acid, and trifluoroacetic acid were purchased from Merck (Darmstadt, Germany). 1-Hydroxybenzotriazole hydrate, dimethyl sulfoxide (DMSO), ammonium bicarbonate, formic acid, Nonidet P-40 substitute (NP-40), super-DHB, NaOH, and tosyl phenylalanyl chloromethyl ketone (TPCK)-treated trypsin from bovine pancreas were purchased from Sigma-Aldrich (St. Louis, MO, USA), 1-ethyl-3-(3-(dimethylamino)propyl)carbodiimide hydrochloride from Fluorochem (Hadfield, Derbyshire, UK), and HPLC SupraGradient acetonitrile (ACN) from Biosolve (Valkenswaard, the Netherlands). Recombinant peptide-*N-*glycosidase F (PNGaseF) was obtained from Roche Diagnostics (Mannheim, Germany). Phosphate-buffered saline (PBS) was made in-house, containing 5.7 g/L Na_2_HPO_4_⋅2H_2_O, 0.5 g/L KH_2_PO_4_, and 8.5 g/L NaCl.

For specifically the UPLC-fluorescence analysis of the 2-aminobenzamide (2-AB) labeled glycans, ultra-pure deionized water was generated by the Millipore Synergy Ultrapure Water Purification System, maintained at ≥18.2 MΩ at 25°C (Merck Millipore, Billerica, MA, USA), whereas formic acid was purchased from Merck, ethanol from Carlo Erba Reagents (Val de Reuil, France) and 2-AB, DMSO, 2-picoline borane, and ACN from Sigma-Aldrich (St. Louis, MO, USA).

### Samples

**Figure 1:**
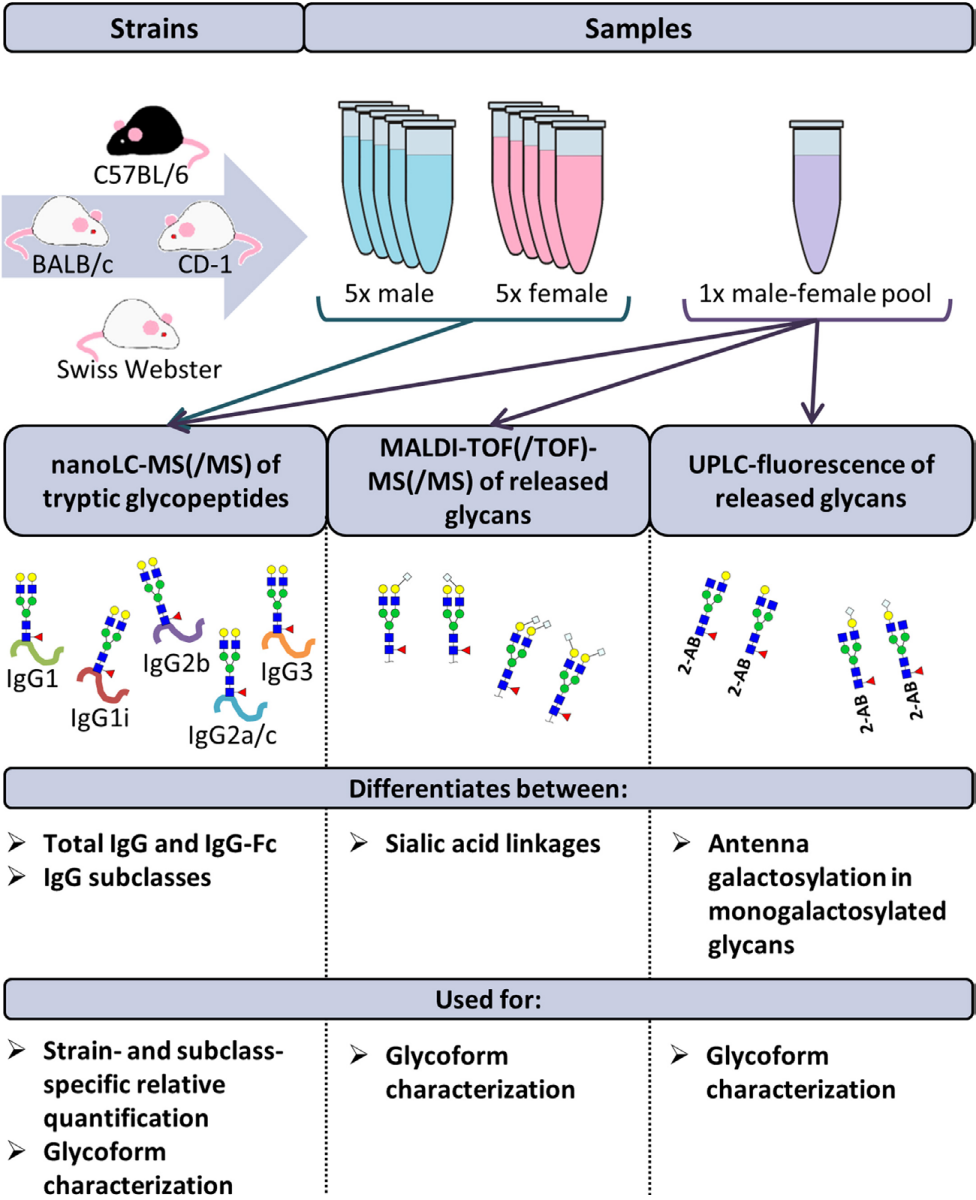
**Schematic representation of the study design**. The immunoglobulin G (IgG) glycopeptides of 40 individual mice were analyzed by nanoliquid chromatography mass spectrometry (nanoLC-MS(/MS)). The mice originated from the strains BALB/c, C57BL/6, CD-1, and Swiss Webster. Of each strain, five male and five female mice were included. Based on these analyses, relative quantification of the glycoforms was performed in a strain- and subclass-specific way. In addition, released IgG glycans of four pooled plasma samples (one male and one female) of the same strains were analyzed by nanoLC-MS(/MS), matrix-assisted laser desorption/ionization time-of-flight (MALDI-TOF(/TOF))-MS(/MS) and ultraperformance liquid chromatography (UPLC)-fluorescence for further structural elucidation of the glycoforms.

The disodium EDTA-plasma of 40 individual mice was purchased from BioChemed Services (Winchester, VA, USA). The mice, aged between 8 and 12 weeks, originated from the strains BALB/c, C57BL/6, CD-1, and Swiss Webster. Of each strain, five male and five female mice were included in the study. Four pooled disodium EDTA-plasma samples (one male and one female) of the same strains were purchased from Seralab (West-Sussex, UK; Figure 1 (20).

### IgG Isolation from Murine Plasma

Murine IgG was captured from 2 μL (for the glycopeptide workflow) or 100 μL (for the released glycan workflow) plasma, using, respectively, 15 or 500 μL protein G affinity beads (GE Healthcare, Uppsala, Sweden) in 100 or 1,000 μL PBS. Proteins were allowed to interact with the beads while shaken for 1 h, after which the beads were washed three times with PBS and three times with water. IgG was eluted in 100 μL 100 mM FA, by incubating the beads 15 min at room temperature with agitation. Eluates were dried for 2 h in a vacuum concentrator at 60°C.

### Preparation of Glycopeptides

Glycopeptide analysis was performed for all 40 individual mouse samples and in addition for 6 technical replicates of the 4 pooled samples in a randomized 96-well plate format. Six blanks were included to serve as negative control. Dried IgG samples (between 3 and 5 μg based on SDS-PAGE gel analysis; Figure S1 in Supplementary Material) were dissolved in 40 μL 25 mM ammonium bicarbonate (pH 8) with 1 μg TPCK-treated trypsin and incubated for 17 h at 37°C. Before nanoLC-MS analysis, all samples were diluted 20 times in ultrapure water.

### Preparation of Released Glycans

Released glycan analysis was performed for the four pooled samples. The complete sample preparation was carried out in triplicate. Dried IgG samples (between 150 and 250 μg based on SDS-PAGE-gel analysis; Figure S1 in Supplementary Material) were dissolved in 20 μL water and 40 μL 2% SDS and incubated for 10 min at 60°C. *N*-Glycans were released by adding 40 μL release mixture (2 mU PNGase F and 2% NP-40 in 2.5× PBS) and incubating for 17 h at 37°C.

Prior to MALDI-TOF-MS analysis, sialic acids were stabilized in a linkage-specific way by ethyl esterification (29). Two microliters of the released glycans were added to 20 μL derivatization reagent (250 mM 1-ethyl-3-(3-(dimethylamino)propyl)carbodiimide and 250 mM 1-hydroxybenzotriazole in ethanol) and incubated for 1 h at 37°C. Twenty microliters of ACN were added and derivatized glycans were enriched by cotton hydrophilic-interaction liquid chromatography (HILIC)−solid-phase extraction (SPE) as described before and eluted in 10 μL water (29, 31).

Prior to UPLC analysis, the released glycans were labeled at the reducing end with 2-AB. Ninety microliters of the released glycans was dried and reconstituted in 50 μL of water. The labeling mixture was freshly prepared by dissolving 19.2 mg/mL 2-AB and 44.8 mg/mL 2-picoline borane in DMSO and glacial acetic acid (70:30, v/v). Twenty-five microliters of labeling mixture were added to each sample in a 96-well plate format, and the plate was sealed using adhesive tape. Samples were mixed by a 10-min shaking step, followed by 2 h incubation at 65°C. After incubation, samples were left to cool down to room temperature for 30 min. The samples (in a volume of 75 μL) were mixed with 700 μL of cold 100% ACN. Free label and reducing agent were removed from the samples using HILIC-SPE on a 0.2 μm GHP filter plate (Pall Corporation, Ann Arbor, MI, USA). Solvent was removed by a vacuum manifold (Millipore Corporation, Billerica, MA, USA). All wells were prewashed using 200 μL of 70% ethanol, followed by 200 μL of water and equilibrated with 200 μL of cold 96% ACN. The samples were loaded onto the GHP filter plate and incubated for 2 min before the vacuum application. The wells were subsequently washed 5 times using 200 μL of cold 96% ACN. The last washing step was followed by centrifugation at 1,000 rpm for 5 min. Glycans were eluted two times with 90 μL of water after 15 min of shaking at room temperature followed by centrifugation at 1,000 rpm for 5 min. The combined eluates were stored at −20°C until usage.

### Nanoliquid Chromatography Mass Spectrometry of Glycopeptides

The 20 times diluted tryptic digests of the IgG samples were separated with an Ultimate 3000 RSLCnano system (Dionex/Thermo Scientific, Breda, the Netherlands) equipped with an Acclaim PepMap 100 trap column (100 μm × 20 mm, particle size 5 μm, Dionex/Thermo Scientific) and an Acclaim PepMap RSLC C18 nano-column (75 μm × 150 mm, particle size 2 μm, Dionex/Thermo Scientific). Two microliters of sample were injected and separated with a gradient from 97% solvent A (0.1% formic acid in water) and 3% solvent B (95% ACN) to 27% solvent B over 15 min, with a flow rate of 700 nL/min. The nanoLC was coupled to a maXis HD quadrupole time-of-flight-MS (q-TOF-MS; Bruker Daltonics) *via* an ESI interface, equipped with the CaptiveSpray and nanoBooster technologies (Bruker Daltonics), using ACN-doped nebulizing gas. Profile spectra were recorded in *m/z* range 550 to 1,800 with a frequency of 1 Hz. The collision energy was 5 eV, the transfer time 130 μs, and the pre-pulse storage 10 μs. The total analysis time per sample was 19 min. The nanoLC system and the q-TOF-MS were operated under Chromeleon Client version 6.8 and otofControl version 4.0.15, respectively. For a closer examination of the glycoforms present, nanoLC-MS/MS was performed on a pooled sample of the undiluted digests of all 40 individual mouse samples. Five microliters of the pooled sample were injected and all putative glycopeptide peaks were selected for MS/MS fragmentation analysis by collision-induced dissociation.

### Matrix-Assisted Laser Desorption/Ionization Time-of-Flight Mass Spectrometry of Released Glycans

MALDI-TOF(/TOF)-MS(/MS) analysis was performed on an UltrafleXtreme (Bruker Daltonics) operated under flexControl 3.3 (Build 108; Bruker Daltonics). One microliter of the enriched ethyl-esterified glycans was spotted on a MALDI target (MTP AnchorChip 800/384 TF; Bruker Daltonics) together with 1 μL 5 mg/mL super-DHB in 50% ACN and 1 mM NaOH. The spots were dried by air at room temperature. For each spot, a mass spectrum was recorded from *m/*z 1,000 to 3,000, combining 10,000 shots in a random walk pattern at 1,000 Hz and 100 shots per raster spot. Prior to the analysis of the samples, the instrument was calibrated using peptide calibration standard (Bruker Daltonics). MALDI-TOF/TOF-MS/MS of the most abundant peaks was performed by laser-induced dissociation.

### Ultraperformance Liquid Chromatography with Fluorescence Detection of Released Glycans

Fluorescently labeled *N*-glycans were separated by HILIC on a Waters Acquity UPLC instrument (Milford, MA, USA) consisting of a quaternary solvent manager, sample manager and a FLR fluorescence detector set with excitation and emission wavelengths of 250 and 428 nm, respectively. The instrument was under the control of Empower 3 software, build 3471 (Waters, Milford, MA, USA). The UPLC system was equipped with a Waters BEH Glycan chromatography column (100 mm × 2.1 mm i.d., 1.7 μm BEH particles). Forty microliters of (80% ACN:20% water) sample were injected and separated with a gradient of 75% solvent B (100% ACN; solvent A:100 mM ammonium formate pH 4.4) to 62% solvent B over 27 min, with a flow of 0.4 mL/min. Solvent B was maintained at 62% for an additional 5 min. Samples were maintained at 10°C before injection, and the separation temperature was 60°C. The system was calibrated using an external standard of hydrolyzed and 2-AB-labeled glucose oligomers from which the retention times for the individual glycans were converted to glucose units.

### Data Processing

For automated relative quantification of the glycopeptides by LaCyTools (version 1.0.1, build 8) (32), the nanoLC-MS files were converted to mzXML files. Chromatograms were aligned based on at least six glycopeptide signals with a signal-to-noise ratio (S/N) above nine, covering the full elution range of the glycopeptides (412–716 s; Table S1 in Supplementary Material). Targeted peak integration was performed on doubly, triply, and quadruply charged species. Twelve chromatographic glycopeptide clusters were defined, one per IgG subclass (IgG1, IgG1i, total IgG2, and IgG3), and within each subclass one per degree of sialylation (0, 1, or 2 sialic acids; Table S2A and Figure S2 in Supplementary Material). Sum spectra were created for these clusters and signals were integrated to include at least 85% of the theoretical isotopic pattern. The actual presence of a glycopeptide was assessed based on the mass accuracy (between −20 and 20 ppm), the deviation from the theoretical isotopic pattern (IPQ; below 25%), and the S/N (above nine) of an integrated signal. Analytes were included for all samples when present in at least 50% of the spectra of one biological group (vendor, strain, and sex). For the glycopeptides that passed analyte curation for total IgG2, new extraction clusters were defined to separate IgG2b glycoforms from IgG2a/c glycoforms (two clusters per analyte; Table S2B in Supplementary Material). Again, glycoforms were included when meeting the requirements described above. Glycopeptide signals were corrected for the actual percentage of isotopic patter integrated, the included charge states were summed per analyte and absolute values were normalized to the total signal intensity per IgG subclass. For the in-depth analysis of compositional features, derived traits were calculated (Table S3 in Supplementary Material; (20, 33)).

For automated relative quantification of the released glycans analyzed by MALDI-TOF-MS, using MassyTools (version 0.1.8.1.) (34), the MALDI-TOF-MS files were converted to text files. Spectra were calibrated based on at least six glycan signals with a S/N above nine, covering the full *m/z* range of the glycans (Table S4 in Supplementary Material). Targeted peak integration was performed for an extensive visually-determined list of glycans, including at least 95% of the theoretical isotopic pattern. The actual presence of a glycan was assessed based on the mass accuracy (between −20 and 20 ppm), the IPQ (below 25%), and the S/N (above nine) of an integrated signal. Analytes were included for all samples when present in at least two-thirds of one of the technical triplicates. Glycan signals were normalized to the total signal intensity.

The chromatographic glycan peaks resulting from the UPLC-fluorescence analysis were integrated using an automatic processing method with the “traditional integration algorithm” after which each chromatogram was manually corrected to maintain the same intervals of integration for all samples. In this way, all chromatograms were separated into 27 peaks and the amount of glycans in each peak was expressed as a percentage of the total integrated area. Assignment of the glycans structures in all major UPLC peaks was performed as described elsewhere (Krištić et al., manuscript submitted).

### Data Analysis

Statistical analysis of the glycopeptide data was performed using R 3.1.2 (R Foundation for Statistical Computing, Vienna, Austria) and RStudio 0.98.1091(RStudio, Inc.). Because of the small sample sizes (between 5 and 40 cases) and the skewing in the distribution of some of the glycosylation traits, non-parametric Mann–Whitney *U* tests were performed to assess sex-, strain-, and subclass-specific glycosylation differences. Multiple testing was accounted for by Bonferroni-correction of the significance threshold (α) per biological question. Differences between the sexes were assessed based on the combined strains (47 tests; α = 0.05/47 = 1.1 × 10^−3^; Table S5 in Supplementary Material), as well as for the individual strains (168 tests; α = 3.0 × 10^−4^; Table S5 in Supplementary Material). Subsequently, the sexes were combined to compare the glycosylation between the different strains (222 tests; α = 2.3 × 10^−4^; Table S6 in Supplementary Material), between the different subclasses of the combined strains (87 tests; α = 5.7 × 10^−4^; Table S7 in Supplementary Material), and between the different subclasses of the individual strains (87 tests each; α = 5.7 × 10^−4^; Table S8 in Supplementary Material).

## Results

### Glycoform Characterization

**Figure 2:**
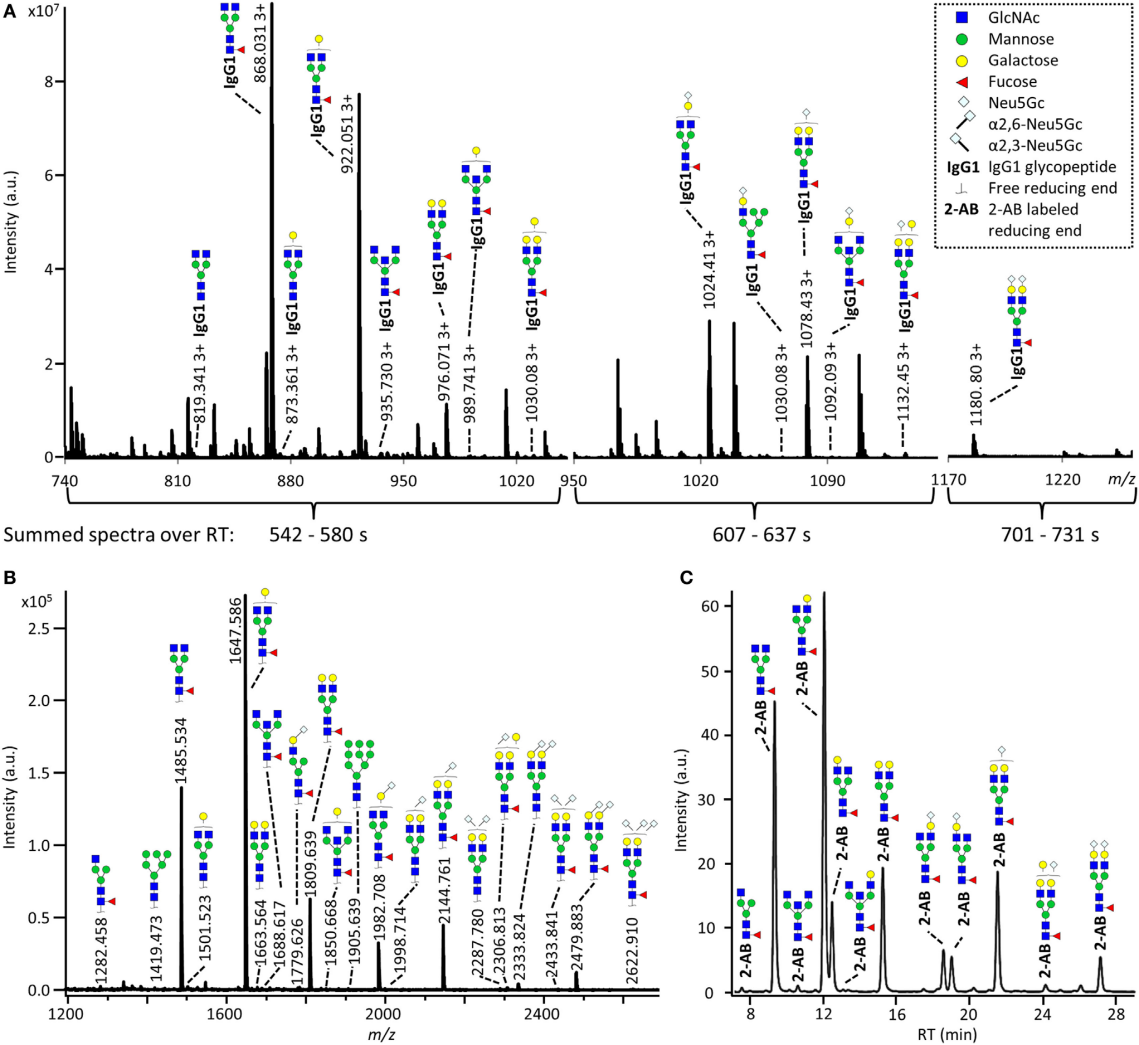
**Glycoforms detected by the three synergetic analysis methods**. Representative data and analytes detected in the analysis of the pooled CD-1 sample, showing **(A)** immunoglobulin G1 (IgG1) fragment crystallizable (Fc)-glycopeptides analyzed by nanoliquid chromatography mass spectrometry (nanoLC-MS), **(B)** the 20 most abundant released glycans of total IgG analyzed by matrix-assisted laser desorption/ionization time-of-flight (MALDI-TOF)-MS after linkage-specific sialic acid derivatization, and **(C)** released glycans of total IgG analyzed by ultraperformance liquid chromatography (UPLC)-fluorescence after 2-AB labeling. Except for the sialic acid linkage in the MALDI-TOF-MS analysis **(B)**, and the antenna galactosylation in the UPLC-fluorescence analysis **(C)**, the monosaccharide linkages were not determined. The proposed glycan structures are based on fragmentation and literature (17, 23, 35, 36).

The IgG Fc-glycosylation of 40 individual mice of four strains (BALB/c, C57BL/6, CD-1, and Swiss Webster) and both sexes (five mice per strain per sex) was analyzed by nanoLC-MS(/MS) of tryptic glycopeptides in a subclass-specific manner. In addition, the strain-specific IgG Fc-glycosylation was studied for four plasma pools of one male and one female mouse per strain (Figures 1 and 2A). A total of 32 different glycan compositions was detected on the combination of IgG subclasses, whereof 27 on IgG1, 22 on IgG1i, 24 on IgG2b, 25 on IgG2a/c, and 21 on IgG3, showing a vast overlap of the glycoforms present per subclass (Table 1; Figures S2–S5 in Supplementary Material). In addition to the nanoLC-MS(/MS) analysis of glycopeptides, the total IgG glycans of the four pooled samples (one of each strain) were enzymatically released and analyzed by both MALDI-TOF(/TOF)-MS(/MS) after sialic acid linkage-specific derivatization and UPLC-fluorescence after 2-AB labeling. The latter two methods allowed the distinction between α2,3- and α2,6-linked sialic acids, and α1,3- and α1,6-antenna galactosylation, respectively (Figures 2B,C).

**Table 1:** **Detected glycoforms per IgG subclass by nanoLC-MS**.

Glycoform composition^a^	Depiction^b^	IgG1^c^	IgG1i	IgG2b	IgG2a/c	IgG3
H2N3F1	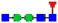	x	x	x	x	
H5N2	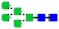	x	x			x
H3N3F1	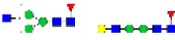	x	x	x	x	x
H3N4	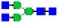	x	x			x
H6N2	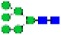	x				
H4N3F1	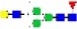	x	x	x	x	x
H3N4F1	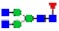	x	x	x	x	x
H4N4	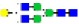	x	x	x	x	x
H5N3F1	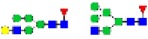		x	x	x	
H4N4F1	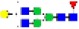	x	x	x	x	x
H5N4	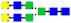	x		x	x	x
H3N5F1	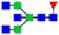	x	x		x	
H4N5	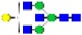					x
H4N3F1G1	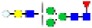	x	x	x	x	x
H4N4G1	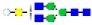		x			
H5N4F1	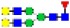	x	x	x	x	x
H4N5F1	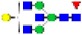	x		x	x	x
H5N3F1G1	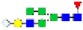	x	x	x	x	
H4N4F1G1	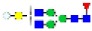	x	x	x	x	x
H5N4G1	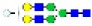	x	x	x	x	x
H6N4F1	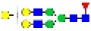	x	x	x	x	x
H5N5F1	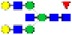	x				x
H6N3F1G1	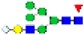	x	x	x	x	x
H5N4F1G1	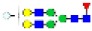	x	x	x	x	x
H6N4G1	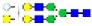	x				
H4N5F1G1	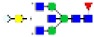	x	x	x	x	x
H5N4G2	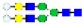			x	x	
H6N4F1G1	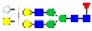	x	x	x	x	x
H5N5F1G1	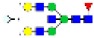	x		x	x	
H5N4F1G1S1	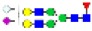			x	x	
H5N4F1G2	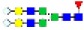	x	x	x	x	x
H5N5F1G2	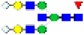	x		x	x	

*^a^H: hexose, N: N-acetylhexosamine, F: fucose, G: N-glycolylneuraminic acid, S: N-acetylneuraminic acid*.

*^b^Symbols used: green circle: mannose, yellow circle: galactose, blue square: *N*-acetylglucosamine, red triangle: fucose, white diamond: *N*-glycolylneuraminic acid, pink diamond: N-acetylneuraminic acid. The proposed glycan structures are based on fragmentation and literature (17, 23, 35, 36). See also Table S9 in Supplementary Material*.

*^c^Peptide sequence of IgG1: EEQFNSTFR, IgG1i: EEQINSTFR, IgG2b: EDYNSTIR, IgG2a/c: EDYNSTLR, IgG3: EAQYNSTFR*.

For all subclasses and in all mice the most abundant glycoforms (approximately 90% of the total area) included diantennary glycans with 0, 1, or 2 galactoses, a core fucose, and 0, 1, or 2 α2,6-linked sialic acids, all in agreement with literature (17, 23). In all samples, the sialic acids proved to be mainly *N*-glycolylneuraminic acid (Neu5Gc) (17, 23), with only the IgG2 isotypes showing the additional presence of *N*-acetylneuraminic acid (Neu5Ac) (IgG2a/b/c glycoform H5N4F1G1S1; H: hexose, N: *N*-acetylhexosamine, F: fucose, G: Neu5Gc, S: Neu5Ac; Table S9 in Supplementary Material). The presence of these minor amounts of Neu5Ac was also registered by MALDI-TOF/TOF-MS/MS analysis (H5N4F1E1Ge1; E: α2,6-linked Neu5Ac, Ge: α2,6-linked Neu5Gc; Table S9 in Supplementary Material). In addition to the α2,6-linked Neu5Gc, the MALDI-TOF-MS method showed α2,3-linked Neu5Gc to be present in the form of H5N4F1Gl1, H5N4Gl1Ge1, and H5N4F1Gl1Ge1 (Gl: α2,3-linked Neu5Gc; Table S9 in Supplementary Material).

Next to the fucosylated diantennary glycans described above, also variants without core fucose, with bisecting GlcNAc or with α1,3-galactosylation on the β-linked-galactoses were detected on mouse IgG-Fc. Bisection was confirmed by nanoLC-MS/MS analysis of the H4N5F1G1 glycoform on IgG1 (Table S9 in Supplementary Material) and in accordance with literature (17, 35). The presence of an extra hexose on diantennary, digalactosylated structures was indicated by nanoLC-MS/MS analysis of the glycan composition H6N4F1G1 on IgG1 (Table S9 in Supplementary Material). In line with literature, this structure as well as H6N4F1 and H6N4G1, were assigned to diantennary structures that carry an α1,3-galactose on one of their antennae (Table S9 in Supplementary Material) (36–38).

Besides diantennary glycans, also monoantennary, high mannose and hybrid structures were detected on the IgG Fc-glycopeptides. While monoantennary glycans were reported before on mouse IgG (17), high mannose and hybrid structures were not. Both H5N2 and H6N2 were detected at the glycopeptides level (Table S9 in Supplementary Material), H8N2 was additionally observed in the MALDI-TOF-MS analysis (Table S9 in Supplementary Material). The presence of hybrid structures was confirmed by nanoLC-MS/MS of H6N3F1G1 on IgG1 (Table S9 in Supplementary Material).

### Sex- and Strain-Specific IgG Fc-Glycosylation

Based on the glycopeptide analysis of the 40 individual mice, it was shown that the two isotypes of IgG1, i.e., IgG1 (UniProt entry P01868) and IgG1i (A0A075B5P4), were simultaneously present in four of the CD-1 mice and five of the Swiss Webster mice, while the others expressed exclusively one of the two forms. In addition, BALB/c and C57BL/6 showed solely the respective presence of IgG1 and IgG1i. IgG2b (P01867/A0A075B5P3), IgG3 (P03987), IgG2a (P01863/P01864) and/or IgG2c (A0A0A6YY53) glycopeptides were observed in all mice, although no distinction could be made between the IgG2a and IgG2c isotypes as they resulted in the same tryptic glycopeptides (Table 1; Table S10 in Supplementary Material).

For the relative quantification of the glycoforms, absolute intensities were normalized per subclass. In addition, derived traits were calculated based on compositional features (Table 2; Table S3 in Supplementary Material). The derived traits per IgG subclass, such as overall galactosylation and sialylation, were compared between the four strains using Mann–Whitney *U* tests. As no difference was found between sexes for any of the testing groups, male and female mice were pooled for all subsequent analyses (Table S5 in Supplementary Material).

**Table 2:** **Derived glycosylation traits**.

Derived trait	Depiction^a^	Description
Total	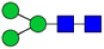	Sum of all glycans
M	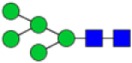	Fraction of high mannose glycans
Hy	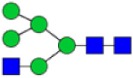	Fraction of hybrid glycans
A1	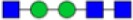	Fraction of N3 glycans (non-hybrid)
A2aF	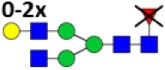	Afucosylation of diantennary glycans
A2B	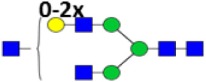	Bisection of diantennary glycans
A2G	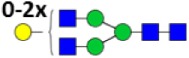	Galactosylation per antenna of diantennary glycans
A2Ga	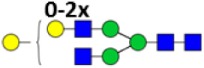	α1,3-Galactosylation per antenna of diantennary glycans
A2GGa	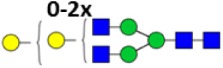	α1,3-Galactosylation per β-galactose of diantennary glycans
A2S	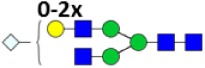	Sialylation per antenna of diantennary glycans
A2GS	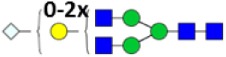	Sialylation per β-galactose of diantennary glycans
S2Sa	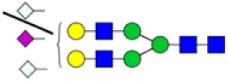	Fraction of Neu5Ac on disialylated, diantennary glycans

*^a^The depictions of the derived traits show the minimally required composition to contribute to a trait. Symbols used: green circle: mannose, yellow circle: galactose, blue square: N-acetylglucosamine, red triangle: fucose, white diamond: N-glycolylneuraminic acid, pink diamond: N-acetylneuraminic acid. For exact calculations per IgG subclass see Table S3 in Supplementary Material*.

**Figure 3:**
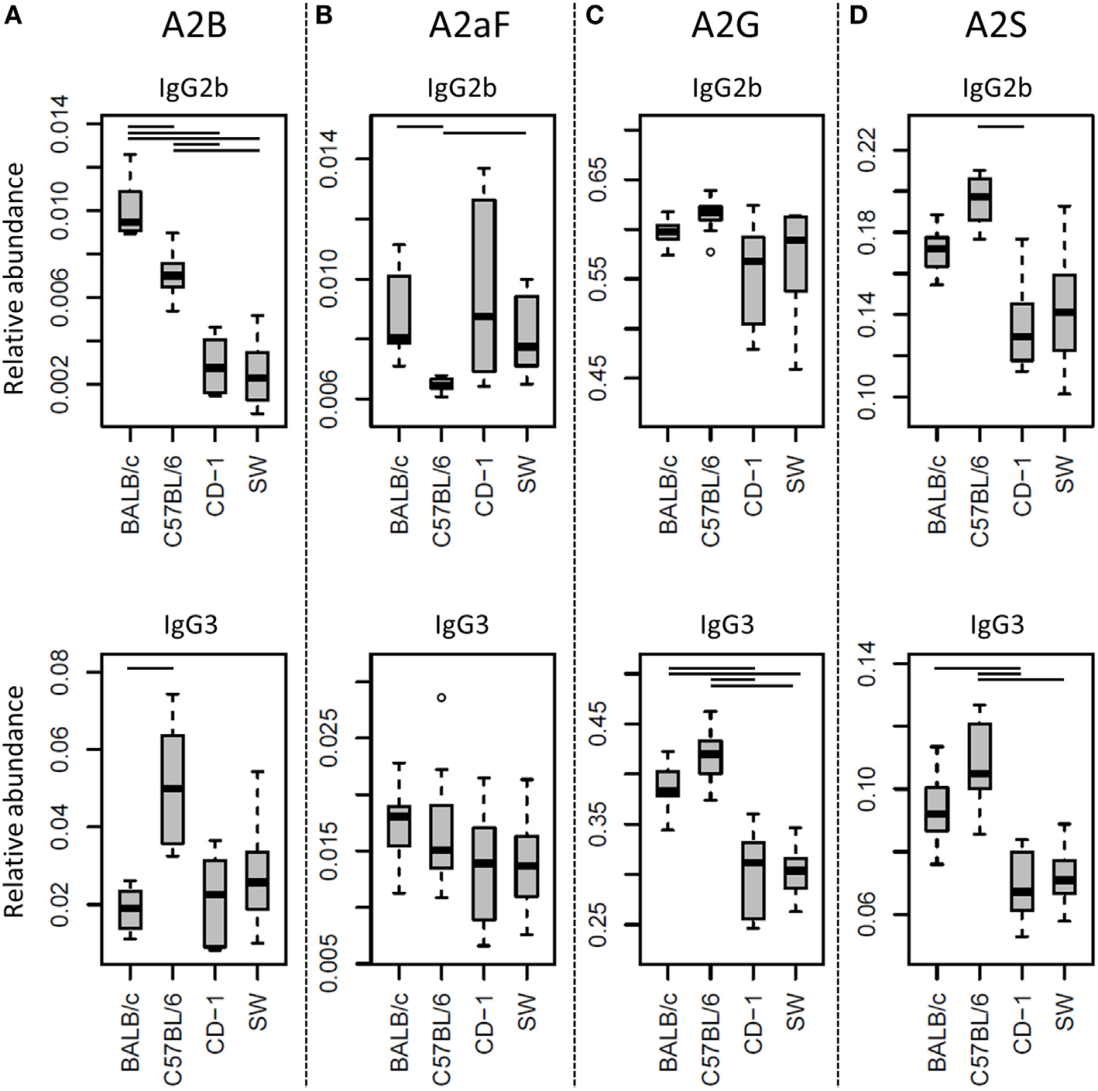
**Differences in the relative abundances of glycosylation traits between strains**. Relative abundances of the derived glycosylation traits **(A)** bisection (A2B), **(B)** afucosylation (A2aF), **(C)** galactosylation (A2G), and **(D)** sialylation (A2S) on immunoglobulin G2b (IgG2b) and IgG3 for the four different mouse strains: BALB/c, C57BL/6, CD-1, and Swiss Webster (SW). Significant differences between strains are indicated by black lines above the boxes. Boxes represent the interquartile range (IQR) and median value, whiskers represent the first and the third quartiles, respectively, minus or plus 1.5 times the IQR.

Many glycosylation traits showed to differ between strains, one of the most pronounced differences being the relative abundance of bisecting GlcNAc on diantennaries (A2B). A2B was low throughout, but clearly higher in BALB/c and C57BL/6 (e.g., IgG2b medians of 1.0 and 0.7%) than in CD-1 and Swiss Webster (0.3 and 0.2%; for statistical test outcomes see Table 3, Table S6 in Supplementary Material, and Figure 3A). This difference was detected for all IgG subclasses except IgG3, where the A2B was found to be similarly low for BALB/c (IgG3 BALB/c: 1.9%, C57BL/6: 5.0%, CD-1: 2.3%, and SW: 2.6%).

**Table 3:** **Fold differences in the derived glycosylation traits between the strains**.

Derived trait^a^	C57BL/6/BALB/c^b^	CD-1/BALB/c	SW/BALB/c	CD-1/C57BL/6	SW/C57BL/6	CD-1/SW
**IgG1**						
A2B	n.d.	**0.22**	**0.27**	n.d.	n.d.	1.22
A2GS	n.d.	**0.68**	**0.75**	n.d.	n.d.	1.10
**IgG2b**						
Hy	0.56	**0.29**	**0.20**	0.53	0.36	0.69
A1	**0.56**	1.33	1.08	**2.36**	1.92	0.82
A2aF	**0.80**	1.09	0.96	1.35	**1.20**	0.88
A2B	**0.74**	**0.29**	**0.24**	**0.39**	**0.33**	0.84
A2S	1.14	0.75	0.82	**0.66**	0.72	1.09
A2GS	1.10	0.80	0.87	**0.73**	0.79	1.08
S2Sa	**0.36**	0.55	**0.37**	1.53	1.03	0.68
**IgG2a/c**						
Hy	0.98	**0.23**	**0.19**	**0.23**	**0.19**	0.83
A2aF	1.27	**0.71**	0.75	**0.56**	0.59	1.05
A2B	1.92	**0.23**	**0.20**	**0.12**	**0.10**	0.87
A2G	**1.39**	1.02	1.06	**0.74**	**0.76**	1.04
A2Ga	**1.93**	0.98	0.98	0.51	**0.51**	1.00
A2S	**2.33**	1.16	1.46	**0.50**	**0.63**	1.27
A2GS	**1.65**	1.09	1.26	0.66	**0.76**	1.15
**IgG3**						
A1	1.18	0.72	0.73	**0.61**	0.62	1.01
A2B	**2.63**	1.19	1.36	**0.45**	0.52	1.14
A2G	1.10	**0.81**	**0.79**	**0.74**	**0.72**	0.97
A2S	1.14	**0.73**	0.77	**0.64**	**0.68**	1.06

*^a^For an explanation of the derived traits see Table 2 and Table S3 in Supplementary Material*.

*^b^Presented values indicate the fold difference between the group medians of strain A/strain B (indicated above each column). Values in bold: statistically significant, n.d.: not determined*.

All strains had low levels of afucosylated glycans, with BALB/c and C57BL/6 having a slightly higher relative abundance of afucosylated diantennaries (A2aF) on IgG2a/c (1.0 and 1.3%) than CD-1 (0.7%; Table S6 and Figure S6 in Supplementary Material). This trend appeared similar for IgG1, IgG1i, and IgG3 (Figure 3B; Table S6 and Figure S6 in Supplementary Material).

Differences in antenna galactosylation (A2G) between the four strains were specifically observed for IgG2a/c and IgG3. On IgG2a/c, galactosylation was high for C57BL/6 (61%), when compared with all other strains (e.g., BALB/c: 44%; Table S6 and Figure S6 in Supplementary Material). On IgG3, high galactosylation was seen in BALB/c and C57BL/6 (38 and 42%), when compared with CD-1 and Swiss Webster (31 and 30%; Figure 3C). UPLC-fluorescence analysis showed furthermore the galactose on the monogalactosylated species to be between 78 and 85% on the α1,6-antenna for H4N4F1 and between 51 and 62% on the α1,6-antenna for H4N4F1G1 (Figure S7 in Supplementary Material). Sialylation (A2S) followed the same behavior as the galactosylation on IgG3 and IgG2a/c, showing IgG3 A2S to be higher for BALB/c and C57BL/6 (9.2 and 11%) than for CD-1 (6.7%), and IgG2a/c A2S to be higher for C57BL/6 (18%) than for all other strains (e.g., BALB/c: 7.5%; Table S6 and Figure S6 in Supplementary Material). In addition A2S on IgG2b was lower for CD-1 (13%) than for C57BL/6 (20%; Figure 3D).

Finally, strain-specific differences were found for the relative abundance of hybrid structures (Hy) on the two isotypes of IgG2, namely a high degree of hybrid structures on IgG2a/c for BALB/c and C57BL/6 (both 0.8%) when compared with CD-1 and Swiss Webster (both 0.2%), and on IgG2b for BALB/c (0.6%) when compared with CD-1 and Swiss Webster (0.2 and 0.1%; Table S6 and Figure S6 in Supplementary Material).

No significant differences were found between the strains for the glycosylation traits on IgG1i, likely due to a reduced statistical power as IgG1i was only present in five of the studied CD-1 and six of the studied Swiss Webster mice. However, the IgG1i derived glycosylation traits followed the trends observed for the IgG2 and IgG3 traits between the strains (Table 3; Table S6 in Supplementary Material). None of the derived glycosylation traits appeared to be different between CD-1 and Swiss Webster.

### Subclass-Specific Glycosylation

Glycosylation differences between subclasses were assessed for the 40 mice of the combined strains using the Mann–Whitney *U* test (Table 4; Table S7 in Supplementary Material). In addition, all glycosylation differences between the subclasses were confirmed by the analysis of the four pooled plasma samples (Figure S8 in Supplementary Material).

**Table 4:** **Fold differences in the derived glycosylation traits between the IgG subclasses**.

Derived trait^a^	IgG1i/IgG1^b^	IgG2b/IgG1	IgG2a/c/IgG1	IgG3/IgG1	IgG2b/IgG1i	IgG2a/c/IgG1i	IgG3/IgG1i	IgG2a/c/IgG2b	IgG3/IgG2b	IgG3/IgG2a/c
Hy	1.09	**0.35**	**0.42**	n.d.	**0.32**	**0.38**	n.d.	1.18	n.d.	n.d.
A1	**2.66**	**0.40**	0.82	**1.89**	**0.15**	**0.31**	0.71	**2.05**	**4.73**	**2.30**
A2aF	0.87	**0.39**	**0.47**	0.77	**0.45**	0.54	0.89	1.20	**1.97**	**1.64**
A2B	**0.39**	**0.20**	**0.24**	0.98	**0.51**	0.62	**2.51**	1.22	**4.95**	**4.04**
A2G	**0.76**	**1.77**	**1.38**	1.04	**2.33**	**1.82**	**1.37**	**0.78**	**0.59**	**0.75**
A2Ga	**3.04**	**2.13**	**1.70**	**2.37**	0.70	0.56	0.78	0.80	1.11	1.39
A2GGa	**3.33**	**1.26**	1.19	**2.05**	**0.38**	**0.36**	**0.62**	0.94	**1.63**	**1.73**
A2S	**0.53**	**1.53**	0.89	**0.78**	**2.90**	**1.67**	**1.46**	**0.58**	**0.51**	0.88
A2GS	**0.83**	0.91	**0.68**	**0.79**	1.10	0.82	0.95	**0.75**	**0.87**	1.16

*^a^For an explanation of the derived traits see Table 2 and Table S3 in Supplementary Material*.

*^b^Presented values indicate the fold difference between the group medians of IgG A/IgG B (indicated above each column). Values in bold: statistically significant, n.d.: not determined*.

**Figure 4:**
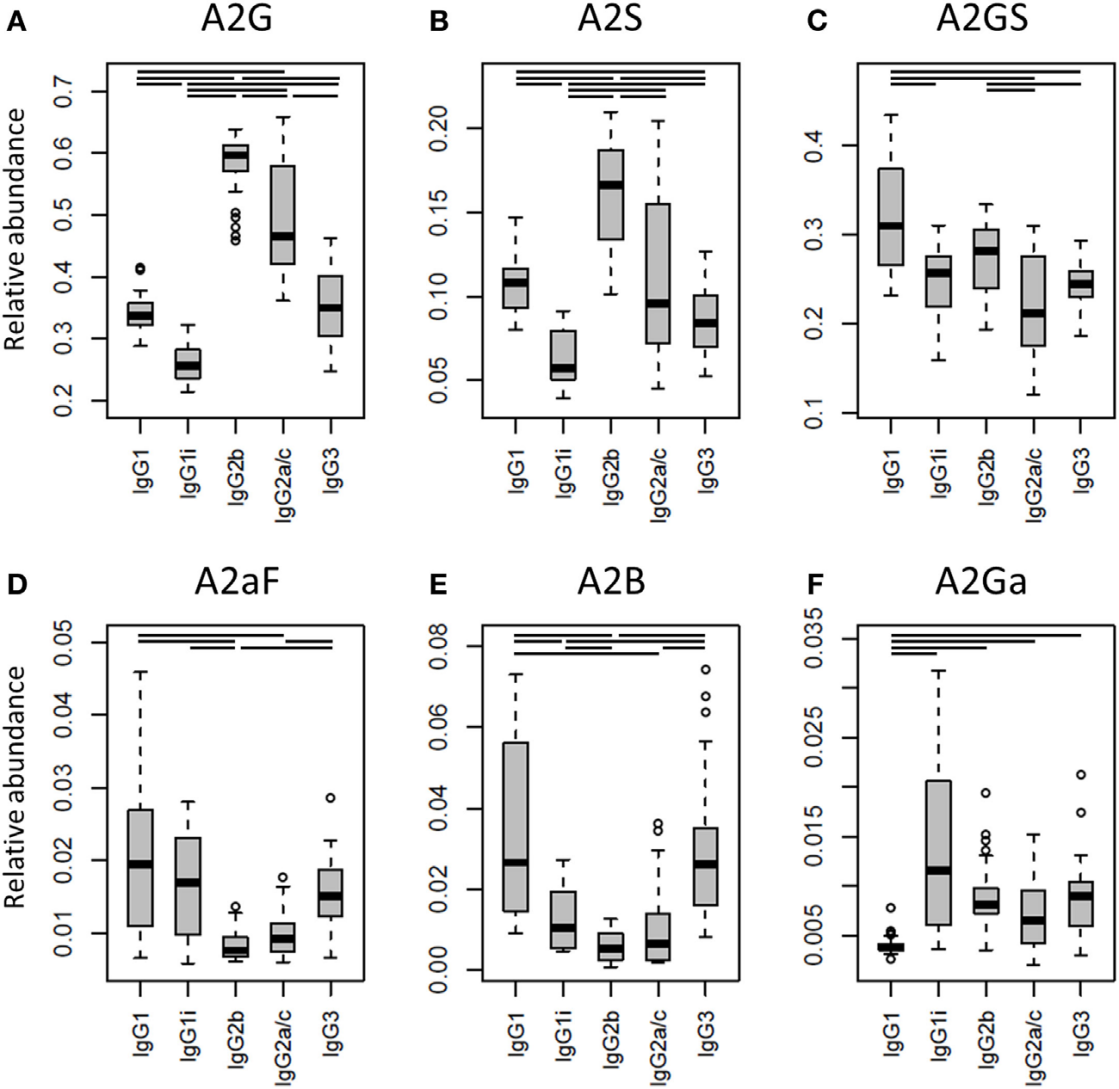
**Differences in the relative abundances of glycosylation traits between the immunoglobulin G (IgG) subclasses**. Relative abundances of the derived glycosylation traits **(A)** galactosylation (A2G), **(B)** sialylation (A2S), **(C)** sialylation per galactose (A2GS), **(D)** afucosylation (A2aF), **(E)** bisection (A2B), and **(F)** α1,3-galactosylation (A2Ga) on the different IgG subclasses studied for the 40 individual mice of all strains. Significant differences between subclasses are indicated by black lines above the boxes. Box and whisker plots as described in Figure 3.

One of the most noticeable differences between subclasses was the level of A2G, which proved highest on IgG2b (median 60%), followed by IgG2a/c (47%), IgG3 (35%), IgG1 (34%), and IgG1i (26%; Figure 4A). A2S showed a similar pattern, being highest on IgG2b (17%) followed by IgG1 (11%), IgG2a/c (9.6%) and IgG3 (8.4%), and IgG1i (5.7%; Figure 4B). Also the sialylation per galactose (A2GS) was not the same for all subclasses, namely higher on IgG1 (31%) and IgG2b (28%) when compared with IgG3 (24%) and IgG2a/c (21%; Figure 4C). This effect could specifically be attributed to BALB/c, which showed low IgG2a/c A2GS (18%) and high IgG1 A2GS (40%; Table S8 in Supplementary Material).

For the overall sample set, the abundance of A2aF was higher for IgG1 (2.0%), IgG1i (1.7%), and IgG3 (1.5%) than for IgG2a/c (0.9%; Figure 4D). Furthermore, A2B, was consistently low on both IgG2 isotypes (IgG2b: 0.5%, IgG2a/c: 0.7%), as well as on IgG1i (1.0%), compared to IgG1 (2.7%) and IgG3 (2.6%; Figure 4E). The presence of α1,3-galactosylation on diantennaries (A2Ga) was relatively constant over all subclasses (IgG2a/c: 0.7%, IgG2b: 0.8%, IgG3: 0.9%, and IgG1i: 1.2%), except on IgG1 (0.4%), on which it was significantly lower than on all others (Figure 4F). This effect was even more pronounced when studied for BALB/c specifically (IgG1: 0.4% and, e.g., IgG2b: 0.9%; Table S8 in Supplementary Material).

## Discussion

In this research we present, to our knowledge, the most in-depth characterization of mouse IgG Fc-glycosylation conducted to date. Next to thorough IgG glycoform characterization, we provide the subclass-specific comparison of the IgG Fc-glycosylation of several commonly used mouse strains. Previous studies into the characterization of mouse IgG glycosylation made use of enzymatically released glycans from isolated IgG, losing both Fc- and subclass-specificity (17, 35). Here, IgG Fc-glycosylation profiling was achieved in a subclass-specific way by analyzing tryptic glycopeptides by nanoLC-MS(/MS). The methodology was reported before, but never applied to multiple mice of different strains and sexes (23). We included in our study five male and five female mice of the commonly used strains BALB/c, C57BL/6, CD-1, and Swiss Webster. On top of the nanoLC-MS(/MS) method, we used two additional techniques for the in-depth characterization of the released IgG *N*-glycans, namely MALDI-TOF(/TOF)-MS(/MS) and UPLC-fluorescence.

### Detected Glycoforms

Consistent with literature, our methods revealed the presence of mainly fucosylated, diantennary glycans with zero to two galactoses and sialic acids (17, 23, 35). The sialic acids were predominantly found as α2,6-Neu5Gc, but the presence of minor amounts of α2,6-Neu5Ac was detected as well. As CMP-Neu5Ac is the precursor for the CMP-Neu5Gc synthesis the presence of this substrate may be assumed in murine plasma cells (39). Next to α2,6-Neu5Gc, also α2,3-Neu5Gc was present in low abundances, although this feature could not be assigned to the Fc-portion specifically, as the sialic acid linkage-differentiation was only achieved on the released glycan level (Figure 1). Furthermore, on the released glycan level, we performed with UPLC-fluorescence a relative quantification of α1,6-antenna galactosylation, when compared with the α1,3-antenna galactosylation for monogalactosylated glycans. The α1,6-antenna showed to be preferably galactosylated, consistent with literature on both human and mouse IgG glycosylation (17, 30). Interestingly, the α1,6-antenna preference was lower for the sialylated H4N4F1G1 when compared with non-sialylated H4N4F1, which might be explained by steric constrains of the α1,6-antenna availability for processing as soon as it is galactosylated (40). On the other hand, this ratio might be influenced by varying distributions of these glycoforms over the subclasses, in combination with unequal IgG subclass abundances.

The presence of afucosylated diantennaries, as well as that of a bisecting GlcNAc on the diantennaries, is well-known for human IgG-Fc (30, 33, 41, 42), but has not yet been reported in an Fc-specific way for mouse IgG before (23). Interestingly, we identified both afucosylation and bisection on all IgG subclasses. The enzyme catalyzing the addition of a bisecting GlcNAc to the β-mannose in the *N*-glycan core, GlcNAc-transferase III, was reported to be expressed in human B lymphocytes (43) and murine brain and kidney tissues (44, 45), making the bisection on mouse IgG-Fc not unimaginable. In addition, both bisection and afucosylation were reported before for glycans released from isolated murine IgG (17).

Whereas α1,3-galactosylation was previously only reported on monoclonal IgG produced in the mouse cell-lines SP2/0 and J558L (36, 37), we here demonstrate its presence on polyclonal murine IgG-Fc. α1,3-Galactosylation is immunogenic in humans, in which the α1,3-galactosyltransferase is not functionally expressed (46). For mice, α1,3-galactosylation is a known glycosylation feature (38).

Next to the diantennary glycan variants described, also high-mannose, hybrid and monoantennary structures were identified specifically on the mouse Fc. Where monoantennary glycans were already reported on murine IgG before, both high mannoses and hybrids were not (17). These glycoforms can be expected as they are precursors of the complex diantennary glycans (47).

### Strain and Subclass Differences

Interestingly, not all IgG subclasses were detected equivalently between the mouse strains and even individuals. We revealed the presence of IgG2b and IgG3 in all mice studied. However, the inbred BALB/c and C57BL/6 mice respectively expressed only IgG1 or IgG1i, while several individuals of outbred CD-1 and Swiss Webster mice showed both isotypes at the same time, which would suggest IgG1 and IgG1i to be allelic variants. This situation was prior reported for IgG2a and IgG2c showing IgG2a to be exclusively present in BALB/c and IgG2c in C57BL/6 (23, 24). However, we were not able to assess this, as the tryptic digestion of IgG2a and IgG2c resulted in the same glycopeptides which were detected in all mice.

When comparing the glycosylation traits between the strains, we observed galactosylation and sialylation to have a lower variation between the individual mice of BALB/c and C57BL/6, when compared with CD-1 and Swiss Webster. As outbred strains are expected to have a wider genetic background than inbred strains, this suggests that there is a strong genetic influence on IgG Fc-glycosylation (13, 14, 48). Next to the genetics, also environmental factors might play a role in the differences between the strains, like exposure to pathogens and health status. For example, the high levels of IgG1 bisection and afucosylation and low levels of IgG1 galactosylation in BALB/c mice, when compared with the other strains in this study, may well represent a more proinflammatory phenotype (28, 49, 50).

The difference in variation between the mouse strains was observed before in the analysis of the murine total plasma *N*-glycome (20). Diantennary, fucosylated glycans in the total plasma *N-*glycome, for healthy humans known to be almost exclusively derived from IgG (21), showed a larger biological variation in the strains CD-1 and Swiss Webster when compared with C57BL/6 and BALB/c. To the contrary, whereas for the total IgG glycans in the total plasma *N-*glycome a clear difference was observed between male and female CD-1 and Swiss Webster mice, this was not the case in the current IgG subclass specific glycopeptide analysis. This might be explained by an interplay between, on the one hand, IgG subclass abundances between the sexes, and on the other hand, glycosylation differences between the IgG subclasses. In addition, hitherto unidentified glycoproteins, other than IgG, that show a pronounced sex specificity may overlap with the IgG glycans in the total plasma *N*-glycome. Other sex-related glycosylation differences observed in the total plasma *N*-glycome study are in glycans that are derived from proteins other than IgG and might indeed have a sex-influenced glycosylation pattern.

Both galactosylation and sialylation of IgG Fc-glycans are established traits involved in the anti-inflammatory activity of IgG (4, 51, 52). On the one hand, sialylation has been reported to dampen immune responses in an antigen-independent way by binding SIGN-R1 (mouse) and DC-SIGN (human) (3, 53), is suggested to be involved in the inhibition of B-cell activation in mice (54) and is in addition able to decrease complement-mediated cytotoxicity *via* C1q binding (55). An increased galactosylation, on the other hand, causes improved binding of mouse IgG1 to the FcγRIIB and subsequent downregulation of proinflammatory activities (4). Of note, the latter phenomenon is observed for IgG1, but not for IgG2a, emphasizing the differences between the IgG subclasses (4).

Another trait known to influence antibody activity in both humans and mice is afucosylation. An increased afucosylation on human IgG1 and murine IgG2a and IgG2b is known to result in an improved binding to specific FcγRs (FcγRIII in humans and FcγRIIB and FcγRIV in mice) and thereby increasing antibody-dependent cellular cytotoxicity (ADCC) (28, 56). Similar to the galactosylation described above, it is relevant to study afucosylation in a subclass-specific way to predict its effect. As IgG subclasses have varying binding affinities for the FcγRs, differential afucosylation can alter the ratio between activating and inhibiting FcγR stimulation. For example, the *in vivo* effect of mouse IgG2b afucosylation on ADCC is larger than that of IgG2a (28).

### Translation of Murine IgG-Fc Glycosylation to the Human System

In terms of glycan structures identified, our mouse data match the glycoforms reported on human IgG-Fc to a large extent, but several pronounced and relevant differences were also observed. One example of this is the earlier-reported difference between the most abundant sialic acid structure, namely Neu5Gc in the mouse and Neu5Ac in man (12, 17, 23, 35), and another is the alternative termination of murine glycans by α1,3-galactosylation instead of (or next to) sialylation. Both factors are important in assessing the suitability of mice for immunological studies, as they might change the interaction between IgG-Fc and effector proteins. For example, it was shown that not the mouse sialic acid-binding Ig-like lectin CD22, but only the human CD22 is able to bind Neu5Ac on intravenous immunoglobulin (12, 57).

Of importance for interpretation of the data is that mouse IgG-subclass nomenclature does not match that of humans, human IgG1 and IgG3 are often directed against protein antigens, while for mice this is the case for IgG2a and IgG2b (26, 58). In addition, human IgG4 is similar to mouse IgG1 in its involvement in responses to allergens and mast-cell binding, and human IgG2 is similar to mouse IgG3 in the recognition of carbohydrate antigens (26, 58, 59). Furthermore, the affinity for FcγRs is not consistent between mouse and human IgG subclasses, as human IgG1 has a broad affinity for all FcγRs, while for the mouse this is achieved by IgG2a and IgG2b (60, 61). In addition, the inhibitory FcγRIIB binds in mice to IgG1, 2a, and 2b, while in human it binds to IgG1, 3, and 4 (22).

The mice we studied were aged between eight and twelve weeks and considered young adults (62). Comparing their IgG2b and IgG2a/c galactosylation to that found previously on IgG1 in healthy human between 20 and 40 years, similar levels were observed (63). The other murine IgG subclasses showed considerably lower levels of galactosylation. The murine IgG2a/c and 2b sialylation, on the other hand, was found to be high in mice, when compared with overall human IgG Fc-sialylation. In addition, the overall bisection and afucosylation were lower on all subclasses in mice than in humans (30, 63). This latter finding might be related to the infrequent immunological challenges lab mice experience. The same phenomenon was observed before in very young children (aged between 0.3 and 4 years), showing low levels of afucosylated IgGs, which increased with age (and likely immunological challenges) (33). In addition, high levels of afucosylated antibodies were observed before in human on antigen-specific antibodies against, amongst others, HIV gp120, human platelet antigen and red blood cells (8–10). Interestingly, the mice showed no differences for the Fc-glycosylation features between the sexes. This is different from what is known for human (young) adult IgG glycosylation, where significant differences were reported for IgG Fc-galactosylation and sialylation (63).

## Conclusion

We report pronounced differences of IgG Fc-glycosylation in mice between both subclass and strains. Especially galactosylation and sialylation showed a large variation in their abundance. In addition, the levels of hybrid structures, α1,3-galactosylation, afucosylation, and bisection, all not previously reported on murine IgG in an Fc-specific way, showed to differ between the subclasses and strains. Noticeable variations were observed for the murine Fc-glycosylation when compared with prior-reported human Fc-glycosylation, for example, in terms of sialic acids present (respectively, Neu5Gc and Neu5Ac in mice and humans). Also levels of glycosylation traits that are recognized as important immune modulators, including galactosylation, sialylation, afucosylation and bisection, deviated between humans and mice. When considering mouse models for immunological research, a careful selection should be made both on the mouse strain and on the IgG subclasses used, taking into account the baseline glycosylation profiles and biological engagement.

## Ethics Statement

The mouse plasmas used in this study were obtained from commercial sources. Within the framework of the manuscript no animal experiments were performed.

## Author Contributions

Conceived and designed the experiments: NH, KR, and MW. Performed the experiments: NH, JK, and AH. Analyzed the data: NH, KR, and JK. Wrote the paper: NH, KR, JK, AH, GL, and MW. Supervised study: GL and MW.

## Conflict of Interest Statement

The authors declare that the research was conducted in the absence of any commercial or financial relationships that could be construed as a potential conflict of interest.
