# Supplementary material for: The N-Glycosylation of Mouse Immunoglobulin G (IgG)-Fragment Crystallizable Differs Between IgG Subclasses and Strains
Source: Front Immunol. 2017 May 31;8:608. doi: 10.3389/fimmu.2017.00608 (PMC5449507; doi:10.3389/fimmu.2017.00608)
Supplement: Supplementary file 1 [file Presentation_1.PDF]

Supplementary figures for:

# The *N*-Glycosylation of Mouse IgG-Fc Differs Between IgG Subclasses and Strains

*Noortje de Haan[1], Karli R. Reiding[1], Jasminka Krištić[2], Agnes L. Hipgrave Ederveen[1], Gordan Lauc[2], Manfred Wuhrer*,[1]*

[1]Center for Proteomics and Metabolomics, Leiden University Medical Center, Leiden, The Netherlands

[2]Glycoscience research laboratory, Genos, Zagreb, Croatia

*Corresponding author: m.wuhrer@lumc.nl

**Figure S1.** SDS-PAGE-gel analysis of captured IgG.

**Figure S2.** Subclass-specific glycoform clustering.

**Figure S3.** Relative intensities of the glycoforms extracted for IgG1 and IgG1i.

**Figure S4.** Relative intensities of the glycoforms extracted for IgG2b and IgG2a/c.

**Figure S5.** Relative intensities of the glycoforms extracted for IgG3.

**Figure S6.** Overview of the strain- and subclass-specific glycosylation in the 40 individual mice.

**Figure S7.** Relative abundances of α1,6-antenna galactosylation on monogalactosylated species.

**Figure S8.** Overview of the strain- and subclass-specific glycosylation in the six technical replicates of the four pooled samples.

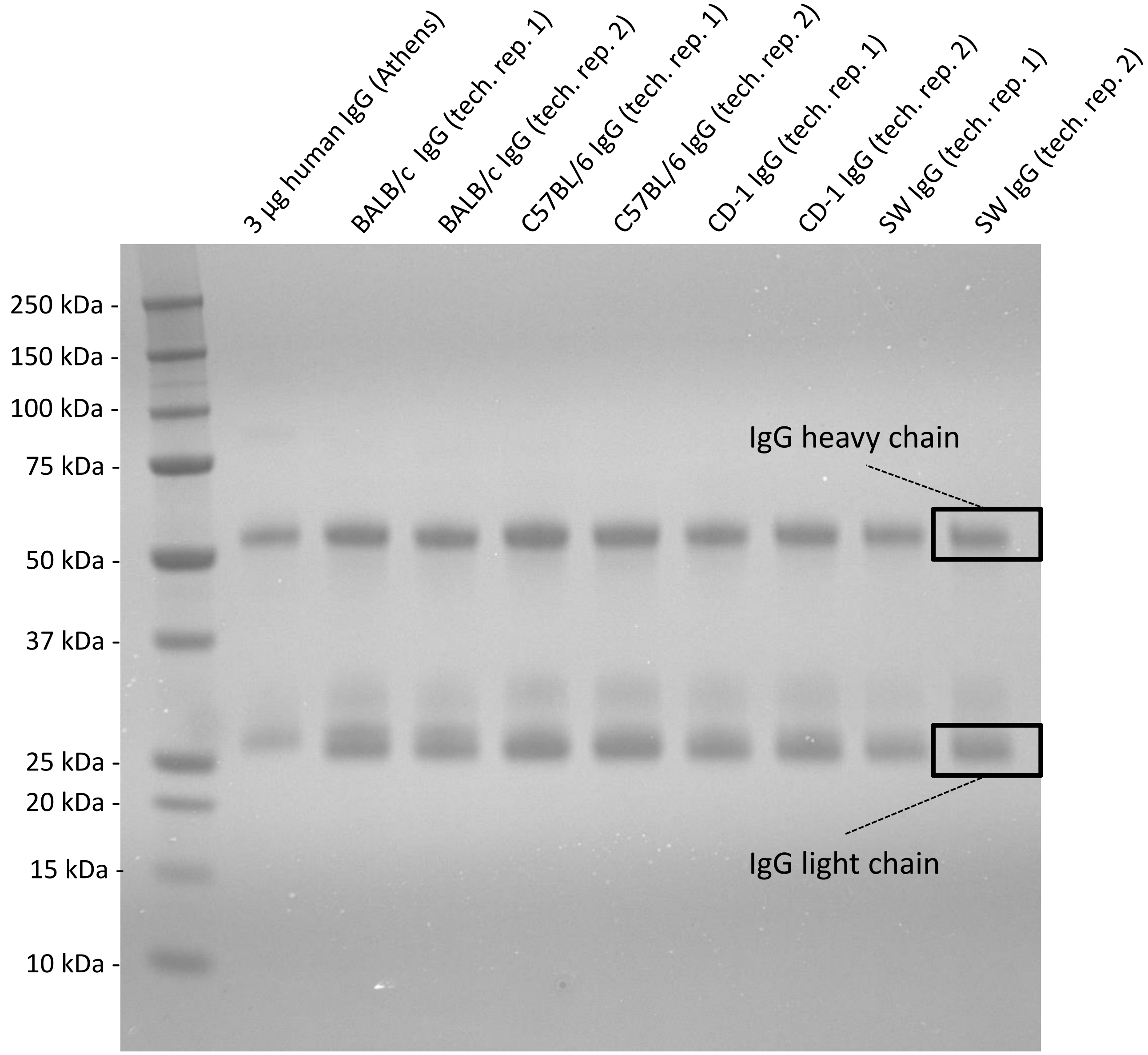


**Figure S1. SDS-PAGE-gel analysis of captured IgG.** For each sample, 2 µL of the 100 µL elualte was brought on gel, corresponding to the IgG isolated from 2 µL murine plasma.

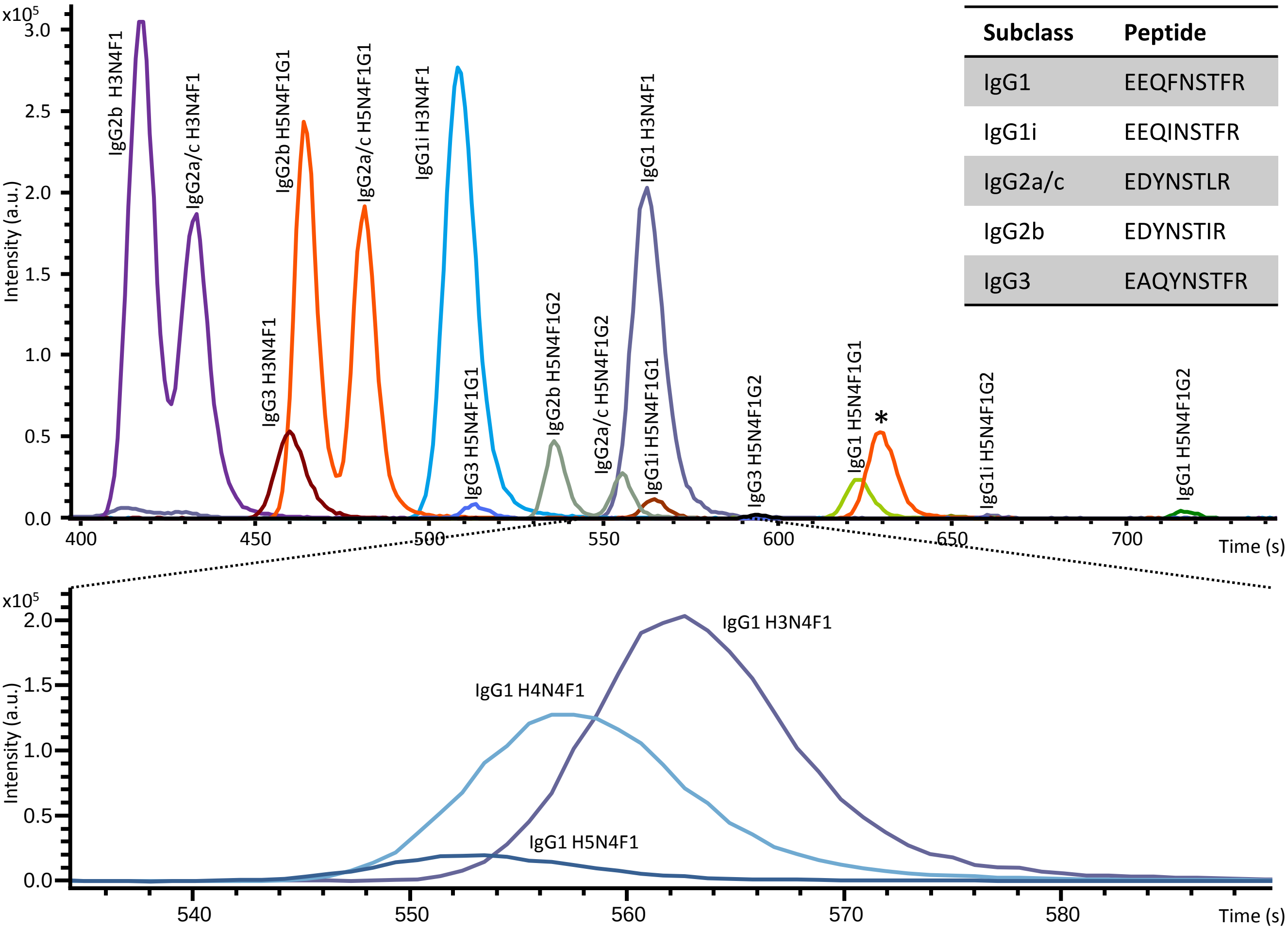


| Subclass | Peptide |
|---|---|
| IgG1 | EEQFNSTFR |
| IgG1i | EEQINSTFR |
| IgG2a/c | EDYNSTLR |
| IgG2b | EDYNSTIR |
| IgG3 | EAQYNSTFR |

**Figure S2. Subclass-specific glycoform clustering.** Representative extracted ion chromatograms of IgG glycoforms of a male Swiss Webster mouse, showing in the upper panel the separation of the glycopeptides based on peptide sequence (IgG subclass) and sialic acid occupancy (zero, one or two). This separation resulted in 15 glycopeptide clusters per sample. The zoom-in shows the influence of the glycoforms within an elution cluster on the retention time of the glycopeptides. *: non-glycopeptide interference, H: hexose, N: *N*-acetylhexosamine, F: fucose, G: *N*-glycolylneuraminic acid.

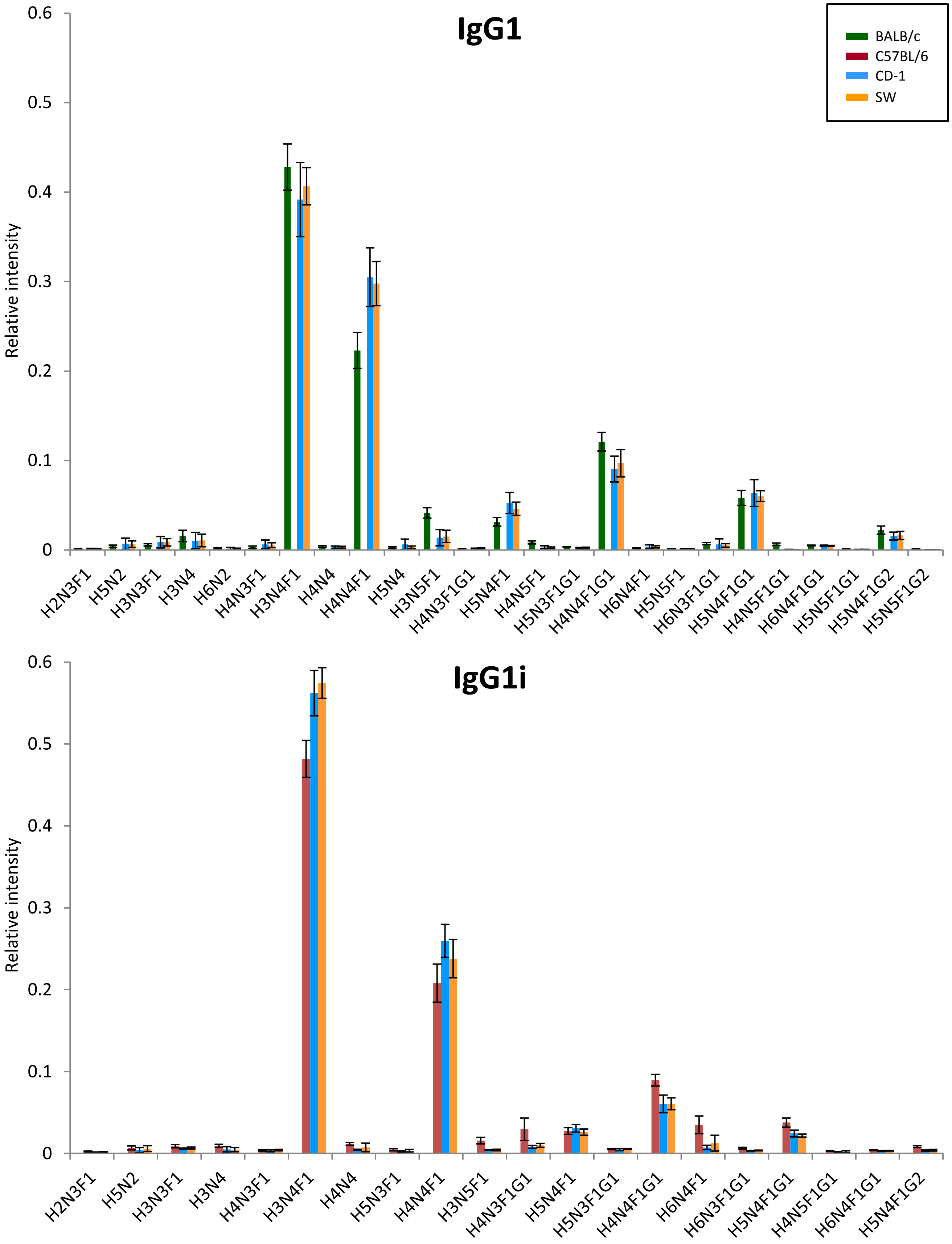


**Figure S3. Relative intensities of the glycoforms extracted for IgG1 and IgG1i.** Averages and standard deviations are calculated over five to ten individual mice per strain. H: Hexose, N: *N*-Acetylhexosamine, F: Fucose, G: Neu5Gc, S: Neu5Ac

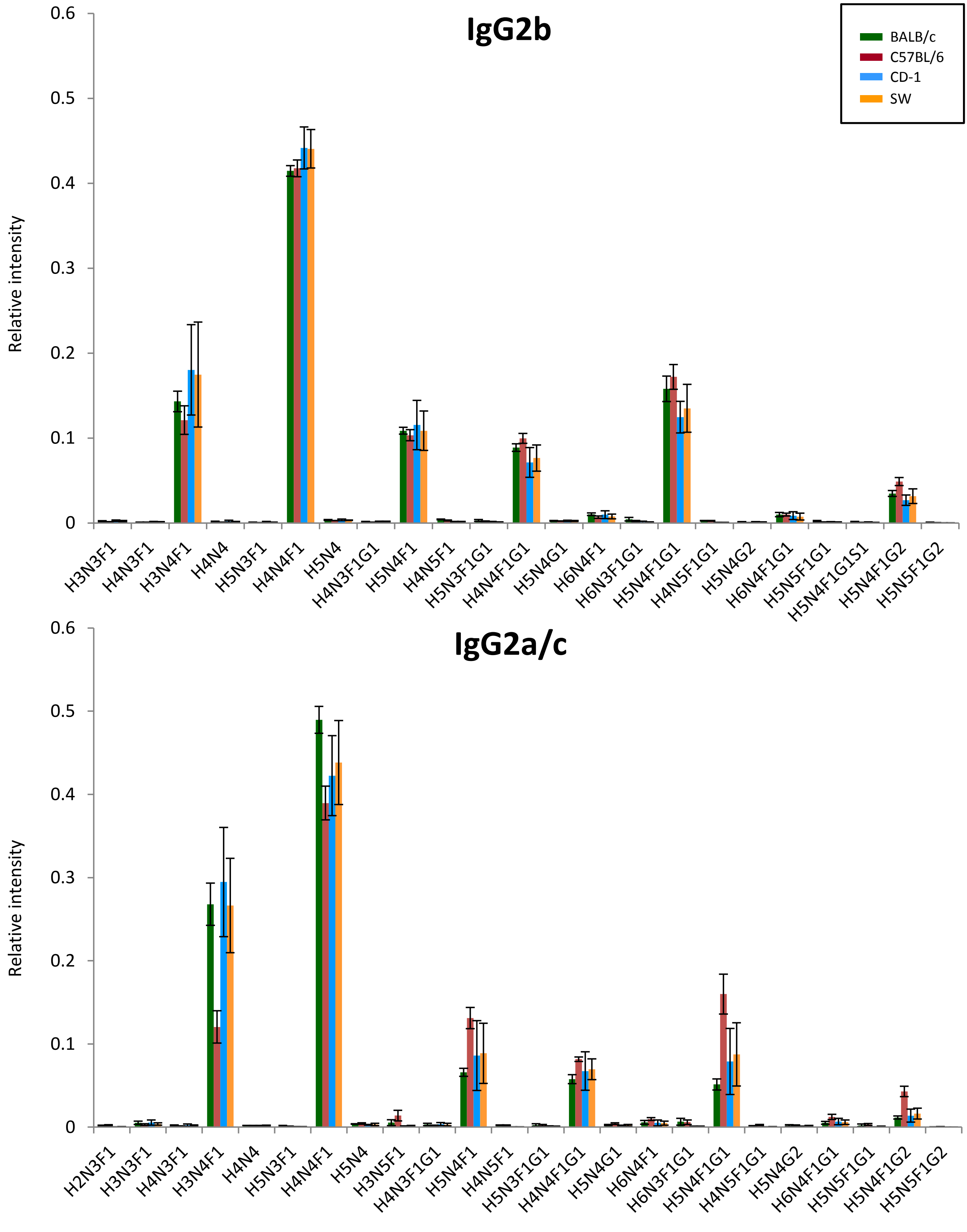


**Figure S4. Relative intensities of the glycoforms extracted for IgG2b and IgG2a/c.** Averages and standard deviations are calculated over five to ten individual mice per strain. H: Hexose, N: *N*-Acetylhexosamine, F: Fucose, G: Neu5Gc, S: Neu5Ac

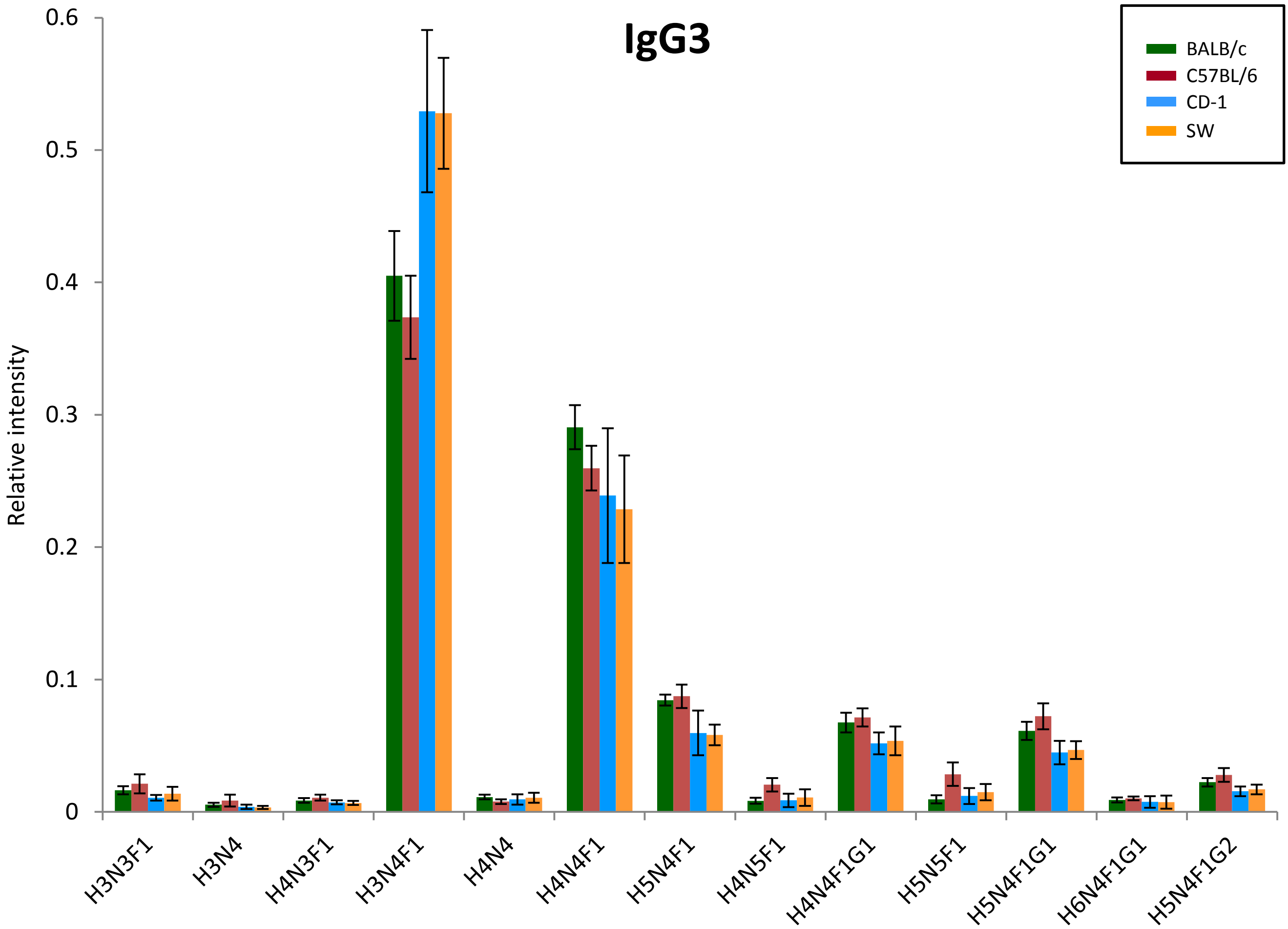


**Figure S5. Relative intensities of the glycoforms extracted for IgG3.** Averages and standard deviations are calculated over five to ten individual mice per strain. H: Hexose, N: *N*-Acetylhexosamine, F: Fucose, G: Neu5Gc, S: Neu5Ac

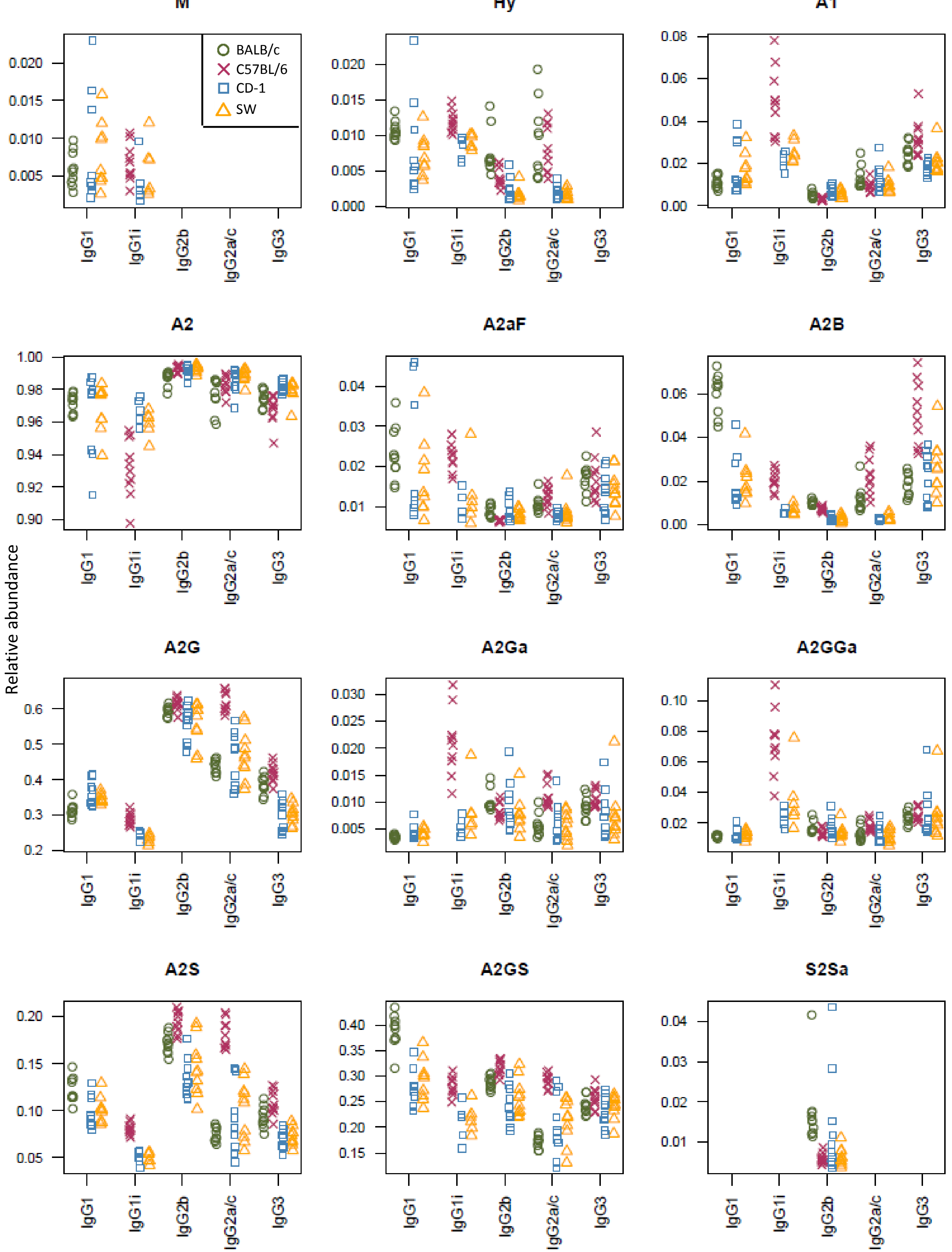


**Figure S6. Overview of the strain- and subclass-specific glycosylation in the 40 individual mice.** Relative abundances of the 12 derived glycan traits on the five subclasses in the individual BALB/c, C57BL/6, CD-1 and Swiss Webster mice. M: fraction of high mannose glycans, Hy: fraction of hybrid glycans, A1: fraction of N3 glycans (non-hybrid), A2: fraction of diantennary glycans, A2aF: afucosylation of diantennary glycans, A2B: bisection of diantennary glycans, A2G: galactosylation of diantennary glycans, A2Ga: α1,3-galactosylation of diantennary glycans, A2GGa: α1,3-galactosylation per β-galactose of diantennary glycans, A2S: sialylation of diantennary glycans, A2GS: sialylation per galactose of diantennary glycans, S2Sa: fraction of Neu5Ac on disialylated diantennary glycans (**Table S3** in Supplementary Material).

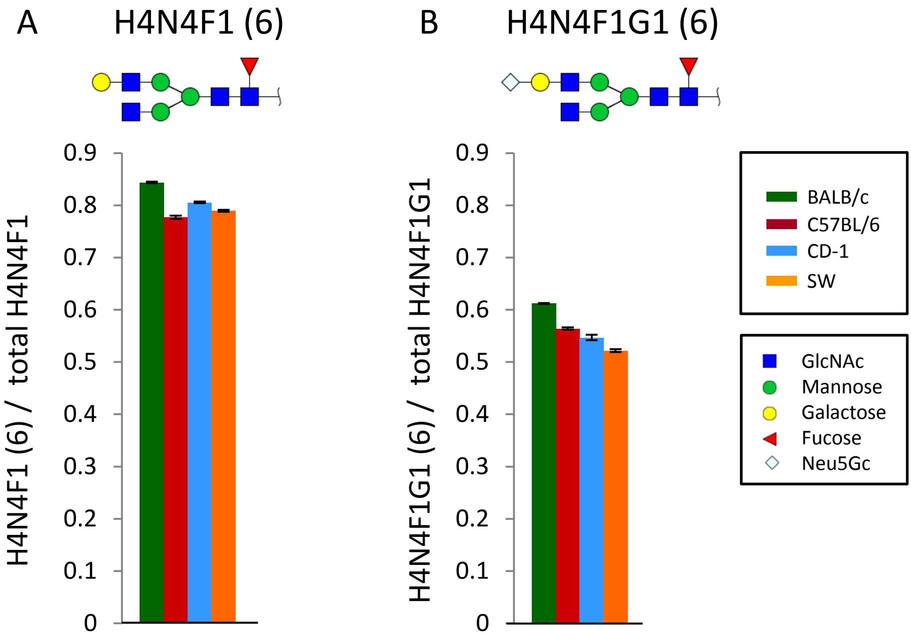


**Figure S7. Relative abundances of α1,6-antenna galactosylation on monogalactosylated species.** Relative abundances of α1,6-antenna galactosylation on (**A**) monogalactosylated diantennary glycans and (**B**) monosialylated, monogalactosylated diantennary glycans. H: hexose, N: *N*-acetylhexosamine, F: fucose, G: *N*-glycolylneuraminic acid, (6): α1,6-antenna galactose.

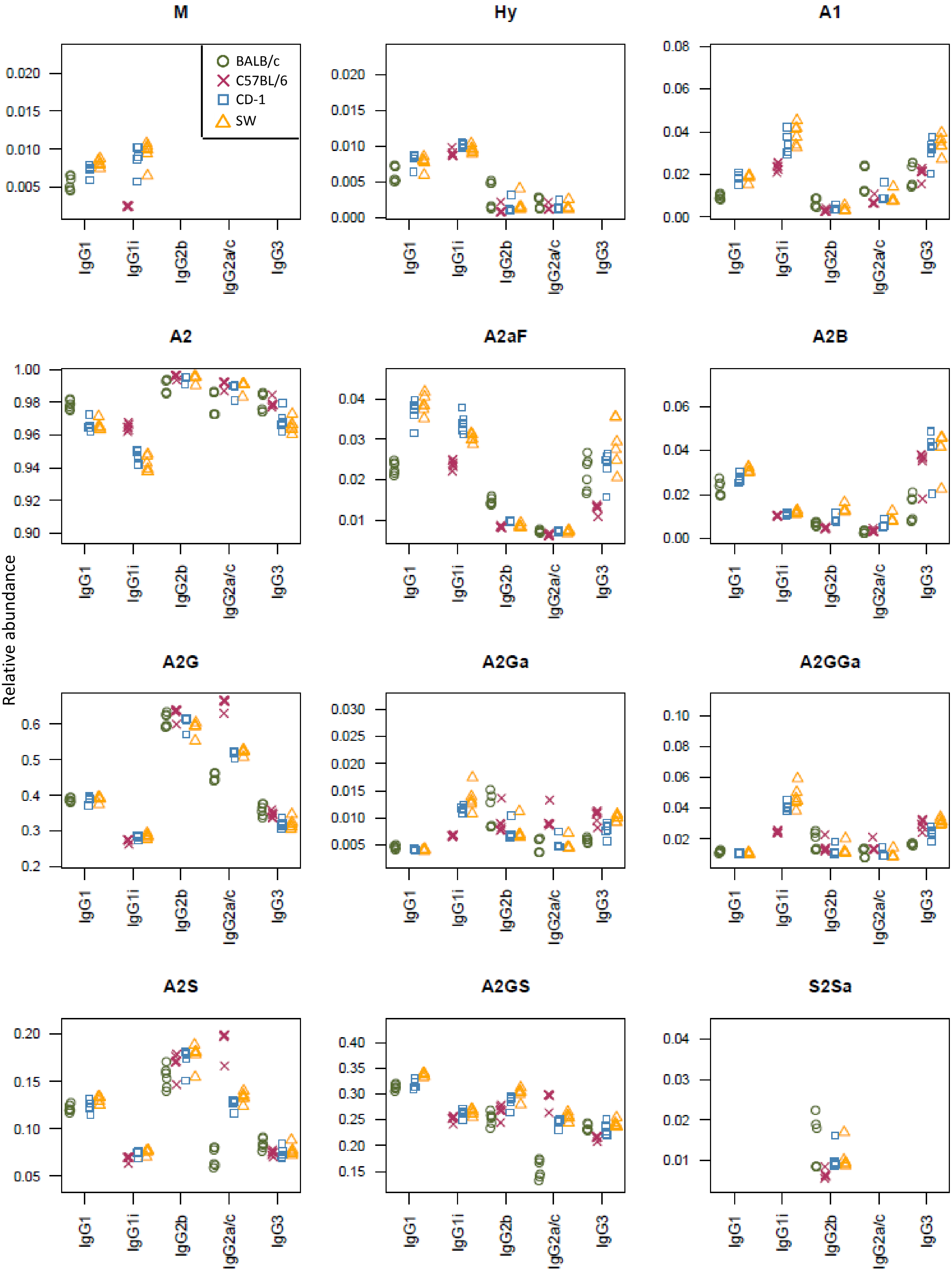


**Figure S8. Overview of the strain- and subclass-specific glycosylation in the six technical replicates of the four pooled samples.** Relative abundances of the 12 derived glycan traits on the five subclasses in the six technical replicates of the pooled BALB/c, C57BL/6, CD-1 and Swiss Webster mice samples. Derived traits as described in **Figure S6** and **Table S3** in Supplementary Material).
